# Multi-Aperture-Based Probabilistic Noise Reduction of Random Telegraph Signal Noise and Photon Shot Noise in Semi-Photon-Counting Complementary-Metal-Oxide-Semiconductor Image Sensor

**DOI:** 10.3390/s18040977

**Published:** 2018-03-26

**Authors:** Haruki Ishida, Keiichiro Kagawa, Takashi Komuro, Bo Zhang, Min-Woong Seo, Taishi Takasawa, Keita Yasutomi, Shoji Kawahito

**Affiliations:** 1 Department of Engineering, Shizuoka University, 3-5-1, Johoku, Nakaku, Hamamatu, Shizuoka 432-8011, Japan; hishi@idl.rie.shizuoka.ac.jp; 2 Research Institute of Electronics, Shizuoka University, 3-5-1, Johoku, Nakaku, Hamamatu, Shizuoka 432-8011, Japan; zhangbo@idl.rie.shizuoka.ac.jp (B.Z.); mwseo@idl.rie.shizuoka.ac.jp (M.-W.S.); ttakasawa@idl.rie.shizuoka.ac.jp (T.T.); kyasu@idl.rie.shizuoka.ac.jp (K.Y); kawahito@idl.rie.shizuoka.ac.jp (S.K.); 3 Graduate School of Science and Engineering, Saitama University, 255 Shimo-Okubo, Sakura-ku, Saitama 338-8570, Japan; komuro@mail.saitama-u.ac.jp

**Keywords:** semi-photon-counting-level CMOS image sensor, random telegraph signal noise, noise reduction, multi-aperture camera, maximum likelihood estimation

## Abstract

A probabilistic method to remove the random telegraph signal (RTS) noise and to increase the signal level is proposed, and was verified by simulation based on measured real sensor noise. Although semi-photon-counting-level (SPCL) ultra-low noise complementary-metal-oxide-semiconductor (CMOS) image sensors (CISs) with high conversion gain pixels have emerged, they still suffer from huge RTS noise, which is inherent to the CISs. The proposed method utilizes a multi-aperture (MA) camera that is composed of multiple sets of an SPCL CIS and a moderately fast and compact imaging lens to emulate a very fast single lens. Due to the redundancy of the MA camera, the RTS noise is removed by the maximum likelihood estimation where noise characteristics are modeled by the probability density distribution. In the proposed method, the photon shot noise is also relatively reduced because of the averaging effect, where the pixel values of all the multiple apertures are considered. An extremely low-light condition that the maximum number of electrons per aperture was the only 2$e^{-}$ was simulated. PSNRs of a test image for simple averaging, selective averaging (our previous method), and the proposed method were 11.92 dB, 11.61 dB, and 13.14 dB, respectively. The selective averaging, which can remove RTS noise, was worse than the simple averaging because it ignores the pixels with RTS noise and photon shot noise was less improved. The simulation results showed that the proposed method provided the best noise reduction performance.

## 1. Introduction

Low light imaging is required in various fields, such as astronomical observation [1], bio-imaging [2], and surveillance [3,4], where high sensitivity cameras are used. Electro-multiplying charge coupled device (EM-CCD) [5,6], high-gain avalanche rushing amorphous photoconductor (HARP) [7], and single photon avalanche diode (SPAD) [8] are examples of well-known high sensitivity image sensors. However, HARP requires a high voltage of several hundred to thousand volts, and EM-CCD and SPAD also require several volts to several tens of volts for avalanche multiplication [6]. In recent years, ultra-low-noise complementary-metal-oxide-semiconductor (CMOS) image sensors (CISs) with a read noise of less than 0.3 $e_{\mathrm{RMS}}^{-}$ based on a high conversion gain floating diffusion have emerged [9,10,11,12,13] and realized semi-photon-counting-level (SPCL) imaging without avalanche amplification that causes excess noise. Unlike CCD image sensors, the source follower amplifier of each pixel in the CIS generates noise with different statistical characteristics. The biggest noise source in the CMOS pixel is random telegraph signal (RTS) noise that is mainly generated by traps of the source follower transistor [14]. It is known that photodiode dark current [15,16,17] and transfer gate [18] also generate RTS noise. RTS noise of the CMOS pixel has become of significant concern because pixels of a certain ratio suffer from this kind of noise [19,20,21]. RTS noise of the source follower is caused by the temporal fluctuation of the threshold voltage $\Delta V_{th}$ due to traps around the channel, and its magnitude is in the order of mV [22,23]. Capture and emission of carriers at the trap in the Si-SiO_2_ interface modulates the electric potential of the channel, evoking $\Delta V_{th}$. This capture and emission process is probabilistic and has a large time constant. Therefore, RTS noise is hardly reduced by the correlated double sampling (CDS) [24]. $\Delta V_{th}$ is expressed by.

(1) $$\Delta V_{th}=\frac{q}{L\cdot W\cdot C_{\mathrm{ox}}}.$$

*q* is the amount of the charges captured or released, *L* and *W* are the gate length and width of MOS transistor, respectively. *C*_ox_ is the capacitance of the gate oxide. In general, in order to reduce the thermal noise of pixel, it is necessary to reduce *L* and the gate area (=*L* · *W*) for higher transconductance and the conversion gain. On the other hand, Equation (1) suggests that RTS noise can become more serious in the high conversion gain pixels.

In the previous work, we have proposed what we call selective averaging [25,26]. This method uses a multi-aperture (MA) camera or a cameras array [27,28,29]. One pixel of a synthesized image is composed of multiple pixels from different image sensors, and the pixels that generate RTS noise are adaptively removed based on the amount of the calculated synthetic noise from the noise measured in the dark condition. Note that the synthetic sensor noise is evaluated pixel by pixel. Application of this method to color imaging [26] and disparity correction with noisy multi-aperture images [30] has been studied. Although it has been proven that the selective averaging method effectively removed RTS noise, the photon shot noise did not decrease efficiently because the number of pixels that were used in reproduction decreased.

In this paper, we propose a noise reduction method based on maximum likelihood estimation [31,32] with an MA camera composed of moderately fast compact imaging lenses and SPCL CISs. To our knowledge, this is the first proposal to remove the RTS noise of SPCL CISs taking advantage of redundancy of the MA camera without any prior of the image and sacrificing any information. The proposed method is applicable to video images because noise reduction is performed frame by frame. Note that one frame is composed of as many images as the apertures, which are simultaneously captured. Firstly, sensor noise is modeled by a probability density function when considering Gaussian noise, RTS noise, and photon shot noise. RTS noise is composed of several discrete levels, and the noise state hops between those levels randomly with a long time constant. Because the MA camera provides multiple pixel values for one pixel in a reproduced image, the RTS noise level can be estimated and the noise is removed. In addition, no pixel value is ignored, unlike the selective averaging method. Consequently, faster imaging optics is virtually realized by the synthetic aperture, and the F-number of much less than unity, which is very difficult to realize with a single-aperture lens, becomes possible. This MA scheme is advantageous in terms of productivity and cost when compared with an expected single-aperture counterpart.

This paper is organized as follows. In Section 2, the MA camera system is explained briefly. Then, probabilistic noise reduction, which is a method based on noise modeling of SPCL CISs and maximum likelihood estimation is mentioned. In Section 3, the proposed method is verified by simulation and compared with conventional methods. In Section 4, several issues of the proposed method are discussed. Finally, Section 5 concludes this paper.

## 2. Probabilistic RTS Noise Reduction with Multi-Aperture Camera

### 2.1. Multi-Aperture Camera

Even if CMOS image sensors had no read noise, they still suffer from photon shot noise in extremely low light conditions because the number of incident photons is often uncontrollable. Charge amplifying image sensors, such as an EM-CCD, can increase the number of photoelectrons. However, it cannot increase the signal-to-noise ratio that is determined by the photon shot noise. In order to reduce the photon shot noise, it is necessary to increase the number of incident photons, which is possible only by collecting more photons with a faster lens. Although the F-number of most lenses is around or more than unity, it must be much smaller, for example, 0.5. However, such ultra-fast lenses could be huge and heavy due to a huge exit pupil and many lens components to correct huge aberrations [33]. A new scheme that realizes an ultra-fast imaging system with smaller optics is desired.

**Figure 1:**
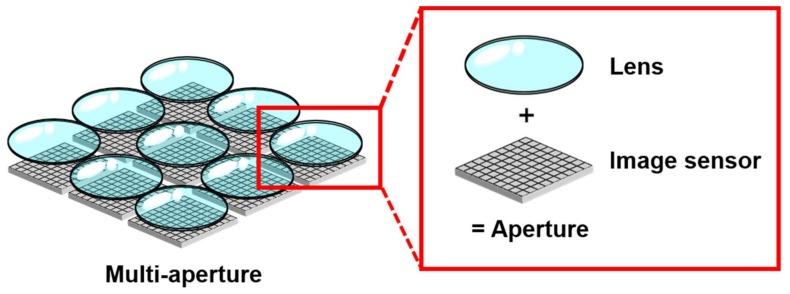
Structure of a multi-aperture camera.

The multi-aperture camera can be an option for the above purpose, which is composed of *M* sets of an imaging lens and an image sensor (Figure 1). The pair of an image sensor and a lens is called an aperture. In the MA system, each aperture observes the same object to obtain redundant images. By summing up *M* images, the signal level becomes *M* times higher. The synthetic F-number, *F_M_*, of the MA camera is given by the following equation.

(2) FM=F0M,

where *F*_0_ is the F-number of the unit lens. Small *F_M_* is achieved with cost-effective, compact, moderately fast lenses. In the past papers, the functionalities of the MA camera have been explored, such as three-dimensional shape measurement from disparity [30], digital refocusing after image acquisition [34], and so on. However, the capability of noise reduction by taking advantage of the redundancy of the MA camera is pursued in this paper.

We have proposed a selective averaging method, in which the pixels that generate RTS noise are adaptively excluded by minimizing the synthesized noise based on the standard deviations measured in darkness before capturing images. The synthesized noise is represented by

(3) $$\hat{m}=\underset{m}{\mathrm{argmin}}\;S_{m}^{2},$$

(4) $$S_{m}^{2}=\frac{1}{m^{2}}\overset{m}{\underset{i=1}{\sum }}\sigma_{i}^{2}\;\;\;\;\;\;\left(1\leq m\leq M\right),$$

where $S_{m}^{2}$ is a combination variance, $\sigma_{i}^{2}$ is an incrementally sorted variance, *m* is the number of the selected apertures, and *M* is the total number of apertures. Normally, as *m* increases, $S_{m+1}^{2}$ becomes smaller than $S_{m}^{2}$ due to the factor of 1/$m^{2}$. However, if some pixels have RTS noise, $S_{m+1}^{2}$ can be greater than $S_{m}^{2}$. Thus, the pixels with RTS noise are automatically removed.

Although the selective averaging is able to remove the RTS noise, the improvement of photon shot noise is sacrificed because a part of pixel values are ignored in averaging. This problem becomes significant, especially when only a few photons are incident in a pixel and ultra-low-noise SPCL CISs whose read noise is almost negligible are utilized.

### 2.2. Noise Modeling of Semi-Photon-Counting-Level Low Noise CMOS Image Sensors

To overcome the above problem, we propose a noise reduction method using maximum likelihood estimation with an MA camera and SPCL CISs [35]. This method is suitable for movies because noise reduction is performed frame by frame. In addition, no prior of the image is assumed. Only modeling of the statistical noise characteristics of the image sensor is necessary. The basic idea is based on the fact that the number of the RTS noise states is limited, for example, 2–5, and those states are measurable before image capturing. If the state of the RTS noise was deducible, the RTS noise could be removed by subtracting its premeasured noise level. Note that, in the MA system, M pixel values are used to reproduce one pixel value in a synthesized image. This redundancy provides the capability to deduce the RTS noise level with a probabilistic estimation method.

Maximum likelihood estimation (MLE) is a classical statistical estimation method, which estimates *λ* from the probability density function $p(x^{\left(1\right)},\ldots ,x^{\left(M\right)}|\lambda )$. In the proposed method, the average number of incident photons, *λ*, for one pixel in the reproduced image is estimated by MLE from M pixel values $\left\{x^{\left(j\right)}\right\}$ that are obtained by the MA camera. MLE is performed in two steps. Step-1: sensor noise for each pixel is modeled as a conditional probability density distribution p(x|λ). Step-2: For every pixel in the reproduced image, the likelihood function L(λ) is calculated and the optimal *λ*, denoted by $\hat{\lambda }$ that gives the maximum likelihood is sought. Here, the likelihood function L(λ) is the product of probability density functions (PDFs), i.e., $p(x^{\left(1\right)}|\lambda )\cdots p(x^{\left(M\right)}|\lambda )$.

(5) $$L\left(\lambda \right)=\overset{M}{\underset{j=1}{\prod }}p\left(x^{\left(j\right)}|\lambda \right),$$

(6) $$\hat{\lambda }=\underset{\lambda }{\mathrm{argmax}}\;L\left(\lambda \right).$$

**Figure 2:**
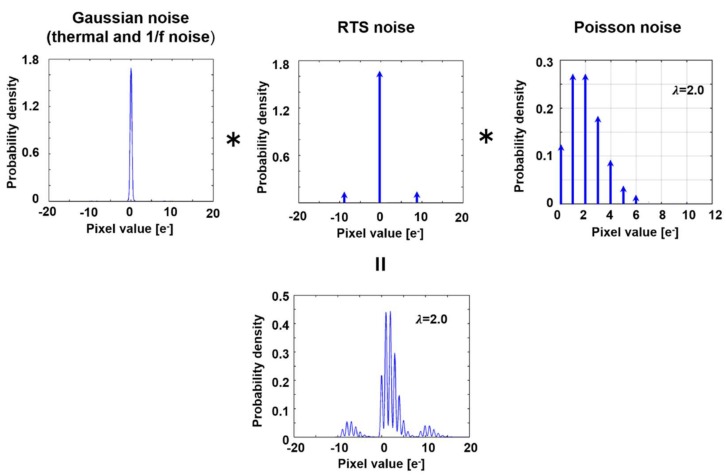
Convolution of three noise components.

Let us consider the stochastic variables, $n_{G},\;n_{RTS},\;\mathrm{and}\;N_{e}$, that correspond to the following noise sources: (1) thermal noise, 1/f noise; and (2) RTS noise of the read circuits; and (3) electron shot noise that was caused by the photogenerated electrons, respectively (Figure 2). The pixel value, *x*, which is also a stochastic variable, is referred to the number of electrons in the floating diffusion of a pixel and denoted by

(7) $$x=n_{G}+n_{RTS}+N_{e}.$$

Note that $n_{G}$ and $n_{RTS}$ are signed real numbers and $N_{e}$ is an integer number (≥0). The PDF of $n_{G}$ is modeled by a Gaussian distribution. The PDF of $n_{RTS}$ becomes weighted one or multiple delta functions, which depict the amount and frequency of RTS noise. Typically, $N_{e}$ obeys a Poisson distribution. Because these stochastic variables are independent and linearly combined, a conditional PDF, $p\left(x^{\left(j\right)}|\lambda \right)$, in terms of the measured pixel value $x^{\left(j\right)}$ of an aperture-*j* for an average number electrons, *λ* (≥0), is given by the convolution of the three PDFs, as follows:

(8) $$p\left(x^{\left(j\right)}|\lambda \right)=\overset{N}{\underset{n=1}{\sum }}\overset{K}{\underset{k=0}{\sum }}\alpha_{n}{}^{\left(j\right)}\cdot \frac{\lambda^{k}}{k!}\exp\left(-\lambda \right)\cdot \frac{1}{\sqrt{2\pi }\sigma^{\left(j\right)}}\exp\left\{-\frac{{\left(x^{\left(j\right)}-k-r_{n}{}^{\left(j\right)}\right)}^{2}}{{\left(2\sigma^{\left(j\right)}\right)}^{2}}\right\}.$$

Here, *n* and *k* are integer numbers, and *λ* is a non-negative real number. The standard deviation of the Gaussian distribution is $\sigma^{\left(j\right)}$. RTS noise is composed of one or multiple discrete levels. $r_{n}{}^{\left(j\right)}$ and $\alpha_{n}{}^{\left(j\right)}$ are the amount and ratio of the *n*-th RTS noise level in electron, and $\left\{\alpha_{n}^{\left(j\right)}|n=1,\;\cdots ,N\right\}$ satisfies $\sum_{n=1}^{N}\alpha_{n}^{\left(j\right)}=1$ for each *j*. *N* is the maximum number of the RTS noise levels. The maximum number of electrons considered is *K*. Note that Equation (8) satisfies the requirements for the PDF, $\int_{-\infty }^{\infty }p\left(x^{\left(j\right)}|\lambda \right)dx^{\left(j\right)}=1$ for any *λ* (≥0).

## 3. Verification by Simulation

**Figure 3:**
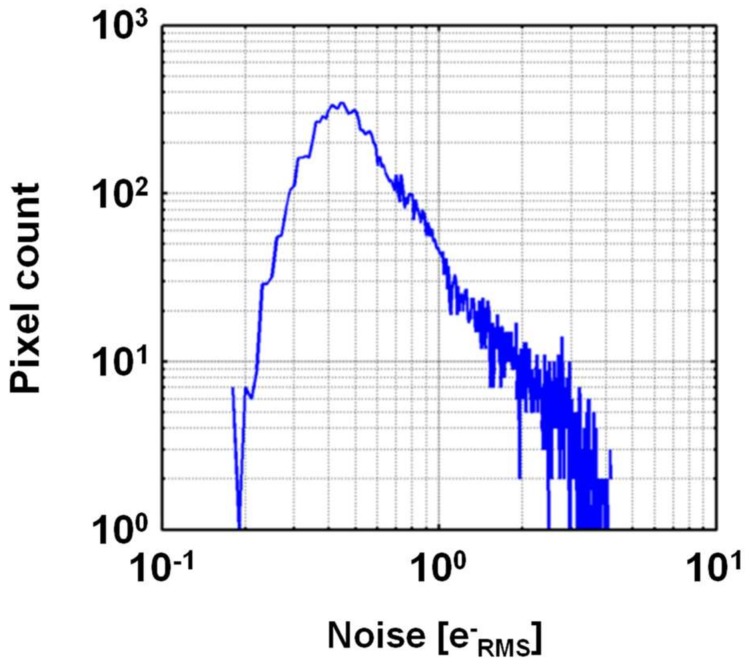
Measured noise histogram of semi-photon-counting-level CMOS image sensor.

The effectiveness of the proposed method was verified by simulation on MATLAB based on measured real noise data. A 3 × 3-aperture camera with an SPCL CIS [12] was assumed. This CIS has a high conversion gain of $220\;\mu V/e^{-}$. In combination with correlated multiple sampling (CMS) [36], extremely low read noise of $0.27{\;e}_{\mathrm{RMS}}^{-}$ was realized. Because the sensor output has a sign bit, negative values that are due to the Gaussian noise and RTS noise of the read circuits are expressed. The sensor was cooled at −10 degree Celsius to suppress dark current shot noise. The measured noise histogram is shown in Figure 3, where RTS and RTS-like noises are included. In order to observe the RTS noise more accurately, the histogram was formed from 5000 dark images. The noise histogram of each pixel was also investigated. The percentage of the pixels without RTS noise, that gave a single peak histogram, was 80.0%. Those for bimodal, trimodal, and tetramodal RTS noise were 18.75%, 1.22%, and 0.03%, respectively. The number of peaks in the histogram was counted when the percentage of the peak exceeded 1% of the primary peak in the evaluation.

**Figure 4:**
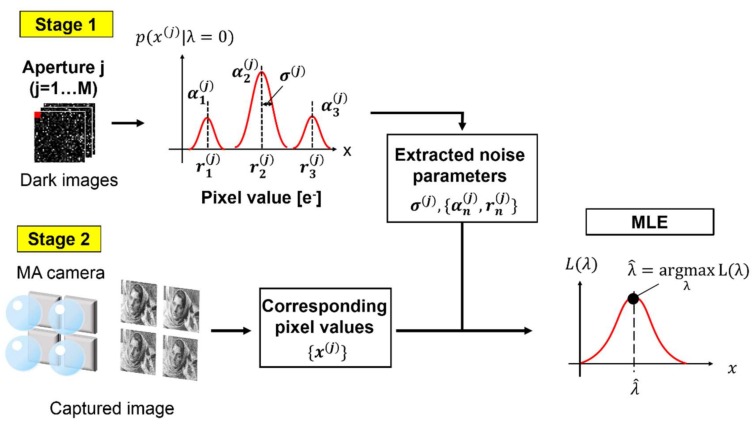
Simulation flow.

**Figure 5:**
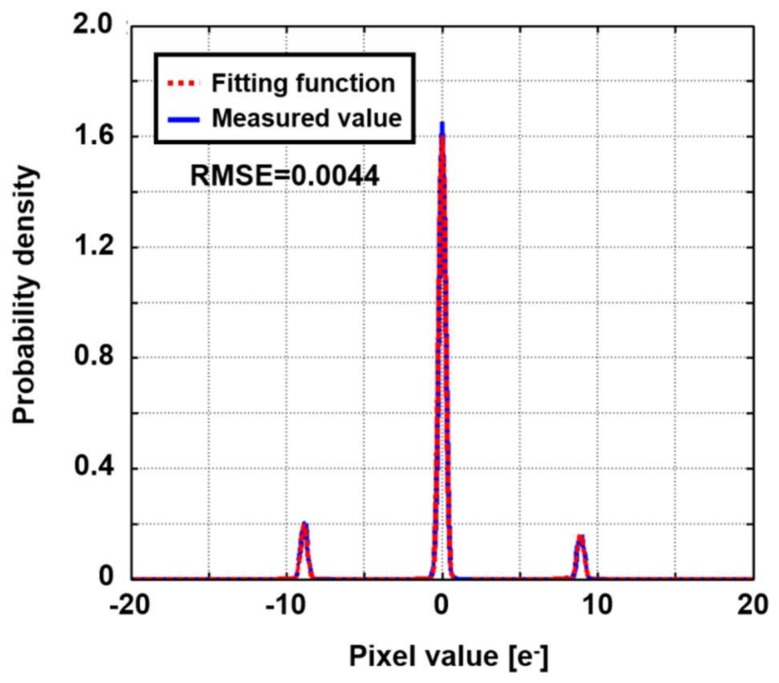
Comparison of fitted and measured noise histograms.

Figure 4 shows a simulation flow of the proposed method, in which measured sensor noise (except electron shot noise caused by dark current and photogenerated electrons) is used. The flow is composed of two stages: (1) noise parameter extraction with dark images and (2) noise reduction of a captured multi-aperture image. The number of effective pixels of the sensor was 31(horizontal) × 510(vertical). Those pixels were reshaped to emulate 3 × 3 apertures, each of which was composed of 40 × 40 pixels. In Step-1, we captured 5000 dark images, which included all kinds of image sensor noise, except for photon shot noise and dark current shot noise. If there are dark current shot noise, multiple peaks whose pitch is equal to one electron is observed. We confirmed that there were no such peaks, which means that no dark current was observed. Because the RTS noise is much larger than one electron, the RTS noise and dark current shot noise are distinguishable. After noise histogram was formed for every pixel, it was fitted to Equation (8). Then, noise parameters, $\sigma^{\left(j\right)}$, {$\alpha_{n}{}^{\left(j\right)},r_{n}{}^{\left(j\right)}\}$ were obtained for each pixel. In Step-2, firstly, a set of *M* noisy images for a ground truth image was generated when considering photon shot noise for the given maximum number of photons, which was generated by MATLAB’s *imnoise* function, and the noise measured in Step-1. In this simulation, the quantum efficiency was assumed to be 100%, namely, the number of incident photons was equal to that of the photogenerated electrons. For every pixel in the reproduced image, *M* corresponding pixel values in the generated images were picked up as $\left\{x^{\left(j\right)}\right\}$ (*j* = 1, ..., *M*). Then, $\hat{\lambda }$ was found by MLE. The search of $\hat{\lambda }$ was performed by nonlinear optimization by a sequential quadratic programming method that was prepared in MATLAB. An initial value was given by selective averaging. Figure 5 shows an example of a fitted histogram for a trimodal pixel. Root mean square error (RMSE) was 0.004 $e_{\mathrm{RMS}}^{-}$. The mean value of RMSE for all of the pixels was 0.012 $e_{\mathrm{RMS}}^{-}$, and peak to peak error was 0.063 $e_{\mathrm{RMS}}^{-}$. Fitted histograms sufficiently matched the measured noise histograms.

**Figure 6:**
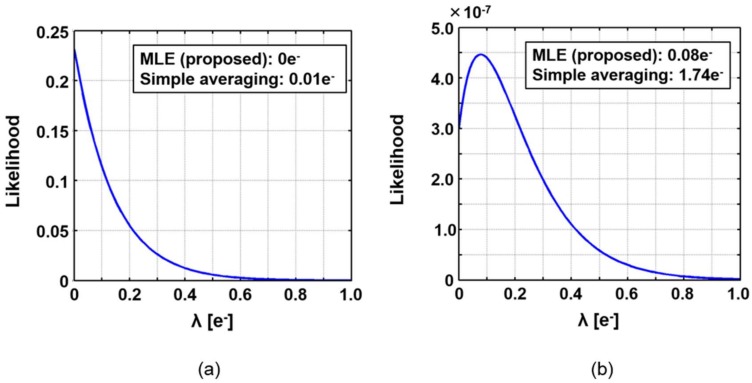
Examples of likelihood functions and estimated pixel values for cases: (**a**) without and (**b**) with random telegraph signal (RTS) noise.

Firstly, the proposed method was applied to dark images where the true value of *λ* should be 0. Figure 6a is an example of the likelihood function for pixels without RTS noise. In this case, the estimated values by both MLE and simple averaging became very close to the true value. On the other hand, the likelihood function for the pixels with RTS noise is shown in Figure 6b. In the simple averaging, the estimated value significantly deviated from the true value. However, in MLE, the estimated value was little affected by RTS noise. 

**Figure 7:**
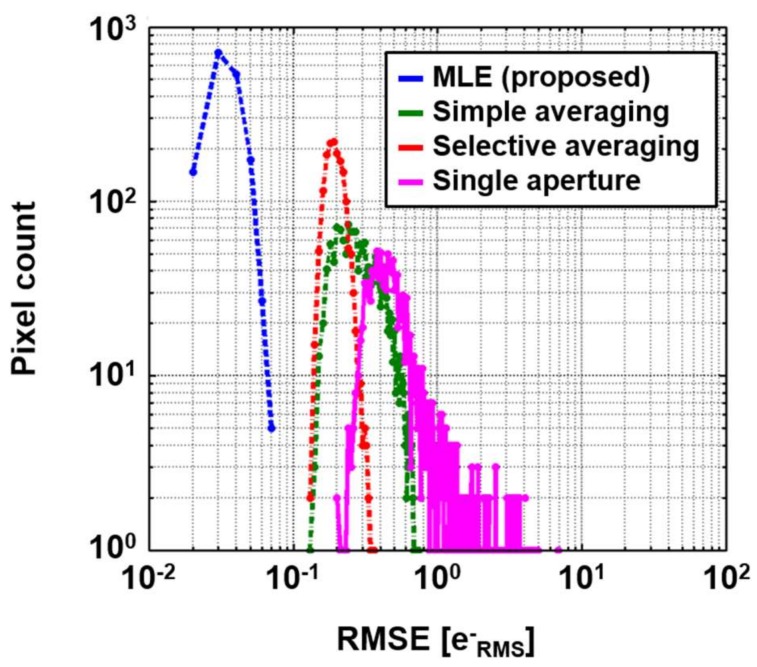
Noise histograms in dark condition.

**Figure 8:**
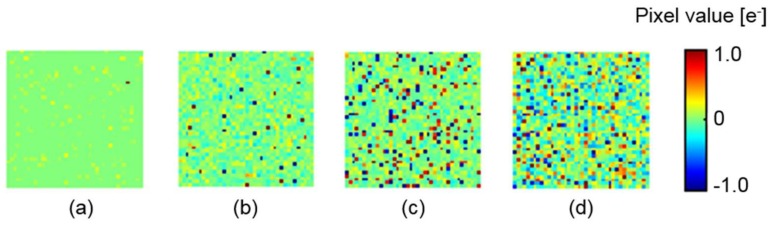
Reproduced dark images by (**a**) the proposed method; (**b**) selective averaging; and (**c**) simple averaging; (**d**) Single aperture image (raw image without noise reduction).

Noise histograms for 5000 dark images are compared in Figure 7, and examples of the processed images are shown in Figure 8. The pixel value is shown by pseudocolor to represent negative values. Those results show that the proposed method is the best noise reduction scheme. In Figure 7, the histogram of a single aperture, which is a reference without any noise reduction, shows the largest peak noise and a long tail caused by RTS noise. Although the peak noise is reduced with the simple averaging, RTS noise still exists. With the selective averaging, RTS noise is effectively removed. However, only 5.94 apertures were selected in the selective averaging because quite a few pixels with the RTS noise were excluded. Therefore, it is expected that photon shot noise is less reduced than in the simple averaging, in which nine apertures are fully utilized. This problem will be discussed later.

In Figure 6a, the estimated pixel values for MLE and the simple averaging are 0 and 0.01 electrons, respectively, in which the true value is 0 electrons. Thus, MLE gives exactly the same as the true value, namely the error is zero, while it is known that simple averaging gives the same variation as MLE if there is only Gaussian noise. It can be because a non-negative constraint for *λ* is assumed in Equation (8). The pixel value was not allowed to be negative. Therefore, it could have been forced to converge to zero. To verify this speculation, we added small shot noise, for example, 0.1 electrons in average. In such a situation, the standard deviations of the estimated pixel values for both MLE and the simple averaging became almost the same, which met the knowledge of statistics mentioned above.

**Figure 9:**
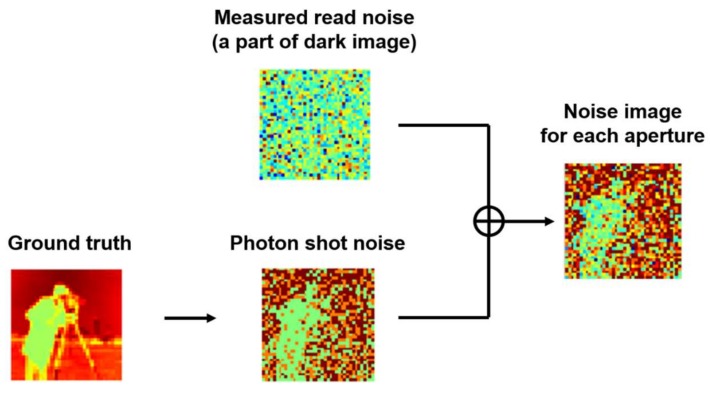
Generating a noisy image from measured read noise and photon shot noise. The signed gray level is represented by pseudocolor. See the scale bar in Figure 10.

Secondly, an extremely low-light condition, where huge photon shot noise existed, was simulated. The maximum number of electrons of the ground truth image was set to 2$e^{-}$ per aperture. The input MA images were created, as shown in Figure 9. Noisy images that include only Poisson noise were generated. Then, measured sensor noise, including Gaussian noise and RTS noise, were added to them. 

**Figure 10:**
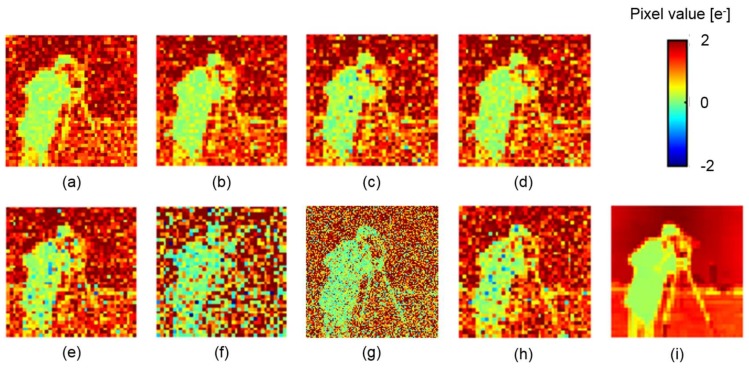
Reconstructed and reference images: (**a**) photon shot noise limited; (**b**) proposed method; (**c**) selective averaging; (**d**) selective averaging (non-negative values); (**e**) simple averaging; (**f**) single aperture; (**g**) single aperture (120 × 120 pixels); (**h**) single aperture (40 × 40 pixels binned from 120 × 120 pixels); and (**i**) ground truth.

Figure 10 shows examples of (a) a photon shot noise limited image, (b–e) the reconstructed images, (f–h) several kinds of raw (single-aperture) image, and (i) the ground truth. The image in Figure 10a includes only photon shot noise without any sensor noise, and the maximum number of electrons is 18$e^{-}$ (=2$e^{-}$ × 9), which should be the best achievable image after noise reduction because the purpose of this paper is the removal of only image sensor noise. The maximum number of photons is rescaled to 2$e^{-}$ in Figure 10a for comparison. Average peak signal-to-noise ratios (PSNRs) and RMSEs of the above images for 100 frames are compared in Table 1. 

In the simple averaging, photon shot noise is reduced. However, RTS noise is very visible, especially on the cameraman in a black cloth. Although RTS noise is significantly reduced with the selective averaging, some pixels with RTS noise still exist. This is because the selective averaging minimizes the synthesized sensor noise. If many pixels for a pixel in the reproduced image have RTS noise, they are averaged instead of ignoring them. As shown in Table 1, the proposed method shows the highest PSNR among three noise reduction methods, and close to the PSNR for the photon shot noise limited case. The remaining difference about 1.4 dB between the proposed method and the photon shot noise limited case can be due to a small Gaussian noise of the SPCL CIS. It is notable that the PSNR for the selective averaging is smaller than that for the simple averaging. Because a part of pixel values was ignored in the selective averaging, in this simulation, the penalty for less improvement of photon shot noise was more significant than the benefit by ignoring the RTS noise pixels. Figure 10d was obtained by replacing the negative pixel values in Figure 10c by zero. The improvement was almost negligible. For comparison, a 120 × 120-pixel image with the same number of the total electrons as that of the MA image was generated (Figure 10g). In this case, the sensor noise became relatively large because the signal level became 1/M for each pixel. Note that the resolution of the ground truth image is 120 × 120 pixels, which is different from that for the other cases. Therefore, PSNR and RMSE were a little worse than those of the single aperture. This image was resized to 40 × 40 pixels with 3 × 3-pixel binning (Figure 10h). Due to the averaging effect, the PSNR and RMSE were improved. However, they were comparable to those of the selective averaging. Consequently, it is shown that the proposed method can remove RTS noise, while photon shot noise becomes close to the photon shot noise limit.

## 4. Discussions

One of the issues of the proposed camera is the removal of disparity. Because multi-aperture images include disparities depending on the lens position and the distance of a subject, they should be removed in the image synthesis. For this purpose, a probabilistic method based on a belief propagation, which is immune to noise, has been studied [30]. However, the signal level that is considered in this paper is extremely low. In such a case, the estimated disparity can be inaccurate, so that the denoised image can become blurry. In future work, this issue should be studied quantitatively.

Although the computation cost was not discussed in this paper, it is very important to implement the proposed method on a commercial camera, in which real-time processing is required. For example, it took 0.78 s and 123.14 s to perform the selective averaging to obtain an initial denoised image and MLE, respectively. In the simulation, MATLAB (R2013a) was run on a workstation (DELL™ PowerEdge T630 Server, Intel Xeon^®^ E5-2698 v3 3.2 GHz × 2, 128 GB memory). A multi-aperture camera with nine apertures and a single-aperture image with 40 × 40 pixels were assumed. At least, the proposed method can be applied to offline or cloud-based post-processing. For real-time processing on a standalone camera, acceleration by parallel hardware should be studied.

The noise parameters of the read circuits are dependent on the temperature [37]. However, they are basically stable in the long term at a moderate temperature. There are two options: one is to keep the sensor temperature constant with a Peltier cooler; the other option is to make a complete table of the noise parameter for different temperatures in the range of possible operating temperature. In this case, the temperature is measured by a thermometer that is embedded in the image sensor during the image capturing.

In the simulation, the variation of transmittance of the imaging lenses was not considered. However, the lenses have a little variation in reality. Furthermore, the transmittance is dependent on the image height due to vignetting, especially in fast lenses, which becomes a problem if the disparity is not negligible. These variations deteriorate our assumption that the incident light intensity is equal for all of the apertures. To compensate these variations, the lens parameters should be measured beforehand and incorporated in the processing. 

The proposed method is very flexible because any noise is modeled by PDF. However, in this paper, classical Gaussian distribution and Poisson distribution are used to formulate the PDF of the image sensor noise. This formulation was suitable for an SPCL image sensor used in this paper. However, for other image sensors, equations that match their noise histogram should be selected, for example, an asymmetric Gaussian, a higher-order Gaussian, and so on.

## 5. Conclusions

In this paper, we simulated noise reduction performance by the maximum likelihood estimation that was applied to a multi-aperture camera using semi-photon-counting-level CMOS image sensors. We modeled the noise characteristics by conditional probability density distributions and confirmed the effectiveness to remove the RTS noise and to reduce the photon shot noise closely to the shot noise limit. In the simulation, a 3 × 3 multi-aperture camera was assumed, and real measured sensor noise, including RTS, was used. We confirmed that the maximum likelihood estimation has the best noise reduction capability when compared with other methods, such as simple averaging and selective averaging. PSNRs (RMSEs) for the single-aperture image, simple averaging, selective averaging, and the proposed method were 2.37 dB (1.36 $e_{\mathrm{RMS}}^{-}$), 11.92 dB (0.49 $e_{\mathrm{RMS}}^{-}$), 11.61 dB (0.51 $e_{\mathrm{RMS}}^{-}$), and 13.14 dB (0.42${\;e}_{\mathrm{RMS}}^{-}$), respectively. The proposed method showed the best noise reduction performance that was close to the shot-noise limited one.

## Figures and Tables

**Table 1:** Peak signal-to-noise ratios (PSNR) [dB] and root mean square error (RMSE) [$e_{\mathrm{RMS}}^{-}$] of the resultant images for each method.

	PSNR [dB]	RMSE [$e_{RMS}^{-}$]
Shot-noise limited	14.52	0.36
MLE (proposed)	13.14	0.42
Selective averaging	11.61	0.51
Selective averaging (non-negative values)	11.76	0.49
Simple averaging	11.92	0.49
Single aperture	2.37	1.36
Single aperture (120 × 120 pixels)	2.20	1.54
Single aperture (40 × 40 pixels binned from 120 × 120 pixels)	11.76	0.49

## References

[1] Tubbs R.N., Baldwin J.E., Mackay C.D., Cox G.C. (2002). Diffraction limited CCD imaging with faint reference stars. Astron. Astrophys..

[2] Niclass C., Sergio M., Charbon E. (2008). A single photon avalanche diode array fabricated in deep-submicron CMOS technology. Design, Automation, and Test in Europe.

[3] Aguilar M., Fay D.A., Ross W.D., Waxman A.M., Ireland D.B., Racamato J.P. (1998). Real-time fusion of low-light CCD and uncooled IR imagery for color night vision. Proc. SPIE.

[4] Blum R.S., Liu Z. (2005). Multi-Sensor Image Fusion and Its Applications.

[5] Jerram P.A., Pool P.J., Burt D.J., Bell R.T., Robbins M.S. Electron Multiplying CCDs. Proceedings of the SNIC Symposium.

[6] Ives D., Bezawada N., Dhillon V., Marsh T. (2008). ULTRASPEC: An electron multiplication CCD camera for very low light level high speed astronomical spectrometry. Proceedings SPIE High Energy, Optical, and Infrared Detectors for Astronomy III.

[7] Tanioka K., Yamazaki J., Shidara K., Taketoshi K., Kawamura T., Ishioka S., Takasaki Y. (1987). An avalanche-mode amorphous Selenium photoconductive layer for use as a camera tube target. IEEE Electron. Device Lett..

[8] Dutton N., Parmesan L., Holmes A., Grant L., Henderson R. 320×240 Oversampled Digital Single Photon Counting Image Sensor. Proceedings of the 2014 Symposium on VLSI Circuits Digest of Technical Papers.

[9] Ma J., Fossum E.R. (2015). Quanta Image Sensor Jot with Sub 0.3e^−^ r.m.s. Read Noise and Photon Counting Capability. IEEE Electron. Device Lett..

[10] Masoodian S., Ma J., Starkey D., Yamashita Y., Fossum E.R. A 1Mjot 1040fps ${0.22e}_{RMS}^{-}$ Stacked BSI Quanta Image Sensor with Cluster-Parallel Readout. Proceedings of the International Image Sensor Workshop (IISW).

[11] Wakashima S., Kusuhara F., Kuroda R., Sugawa S. A linear response single exposure CMOS image sensor with 0.5e^−^ readout noise and 76ke^−^ full well capacity. Proceedings of the 2015 Symposium on VLSI Circuits (VLSI Circuits).

[12] Seo M.-W., Kawahito S., Kagawa K., Yasutomi K. (2015). A ${0.27e}_{RMS}^{-}$ Read Noise 220-μV/e^−^ Conversion Gain Reset-Gate-Less CMOS Image Sensor With 0.11-μm CIS Process. IEEE Electron. Device Lett..

[13] Seo M.-W., Wang T., Jun S.-W., Akahori T., Kawahito S. A ${0.44e}_{RMS}^{-}$ Read-Noise 32fps 0.5Mpixel High-Sensitivity RG-Less-Pixel CMOS Image Sensor Using Bootstrapping Reset. Proceedings of the 2017 IEEE International Solid-State Circuits Conference (ISSCC).

[14] Leyris C., Martinez F., Valenza M., Hoffmann A., Vildeuil J.C., Roy F. Impact of Random Telegraph Signal in CMOS Image Sensors for Low-Light Levels. Proceedings of the 32nd European IEEE Solid-State Circuits Conference.

[15] Virmontois C., Goiffon V., Magnan P., Saint-Pe O., Girard S., Petit S., Rolland G., Bardoux A. (2011). Total Ionizing Dose Versus Displacement Damage Dose Induced Dark Current Random Telegraph Signals in CMOS Image Sensors. IEEE Trans. Nucl. Sci..

[16] Virmontois C., Goiffon V., Robbins M.S., Tauziède L., Geoffray H., Raine M., Girard S., Gilard O., Magnan P., Bardoux A. (2013). Dark Current Random Telegraph Signals in Solid-State Image Sensors. IEEE Trans. Nucl. Sci..

[17] Durnez C., Goiffon V., Rizzolo S., Magnan P., Virmontois C., Rubaldo L. Localization of Dark Current Random Telegraph Signal sources in pinned photodiode CMOS Image Sensors. Proceedings of the International Conference on Noise and Fluctuations (ICNF).

[18] Wang X., Rao P.R., Mierop A., Theuwissen A.J.P. Random Telegraph Signal in CMOS Image Sensor Pixels. Proceedings of the International Electron Devices Meeting (IEDM).

[19] Tega N., Miki H., Osabe T., Kotabe A., Otsuga K., Kurata H., Kamohara S., Tokami K., Ikeda Y., Yamada R. Anomalously Large Threshold Voltage Fluctuation by Complex Random Telegraph Signal in Floating Gate Flash Memory. Proceedings of the International Electron Devices Meeting (IEDM).

[20] Abe K., Sugawa S., Kuroda R., Watabe S., Miyamoto N., Teramoto A., Ohmi T., Kamada Y., Shibusawa K. Analysis of Source Follower Random Telegraph Signal Using nMOS and pMOS Array TEG. Proceedings of the 2007 International Image Sensor Workshop (IISW).

[21] Gonthier P.M., Magnan P. RTS noise impact in CMOS image sensors readout circuit. Proceedings of the International Conference on Electronics Circuits and Systems (ICECS).

[22] Gonthier P.M., Goiffon V., Magnan P. (2012). In-Pixel Source Follower Transistor RTS Noise Behavior under Ionizing Radiation in CMOS Image Sensors. IEEE Trans. Electron. Devices.

[23] Wang X. (2008). Noise in Sub-Micron CMOS Image Sensors. Ph.D. Thesis.

[24] Mendis S., Kemeny S., Gee R., Pain B., Kim Q., Fossum E.R. (1997). CMOS active pixel image sensors for highly integrated imaging system. IEEE J. Solid-State Circuits.

[25] Zhang B., Kagawa K., Takasawa T., Seo M.-W., Yasutomi K., Kawahito S. (2014). RTS Noise and Dark Current White Defects Reduction Using Selective Averaging Based on a Multi-Aperture System. Sensors.

[26] Zhang B., Kagawa K., Takasawa T., Seo M.-W., Yasutomi K., Kawahito S. (2015). Low-light Color Reproduction by Selective Averaging in Multi-aperture Camera with Bayer Color-filter Low-noise CMOS Image Sensors. ITE Trans. Media Technol. Appl..

[27] Naemura T., Harashima H. (1999). A Real-Time System for Image-Based Rendering from a Multi-View Video—Video-Based Rending. Trans. Virtual Real. Soc. Jpn..

[28] Yang J.C., Everett M., Buehler C., McMillan L. A Real-Time Distributed Light Field Camera. Proceedings of the 13th Eurographics Workshop on Rending.

[29] Wilburn B., Joshi N., Vaish V., Talvala E.-V., Antunez E., Barth A., Adams A., Horowitz M., Levoy M. (2005). High Performance Imaging Using Large Camera Arrays. ACM Trans. Graph. (TOG).

[30] Kugenuma K., Komuro T., Zhang B., Kagawa K., Kawahito S. High-sensitivity Imaging Using a Multi-aperture Camera based on Imaging Synthesis with Disparity Compensation. Proceedings of the International Workshop on Advanced Image Technology (IWAIT 2017).

[31] Fisher R.A. (1922). On the mathematical foundations of theoretical statistics. Philos. Trans. R. Soc..

[32] Lehmann E.L., Casella G. (1998). Theory of Point Estimation.

[33] Becker J., Wilhelmus B.H. (1961). Optical Lens System Having a Relative Aperture Larger than f/1. U.S. Patent.

[34] Green P., Sun W., Matusik W., Durand F. (2007). Multi-aperture photography. ACM Trans. Graph. (TOG).

[35] Ishida H., Kagawa K., Seo M.-W., Komuro T., Zhang B., Takasawa T., Yasutomi K., Kawahito S. RTS and photon shot noise reduction based on maximum likelihood estimate with multi-aperture optics and semi-photon-counting-level CMOS image sensors. Proceedings of the IS&T Electronic Imaging, Image Sensors and Imaging Systems 2017.

[36] Seo M.-W., Suh S.-H., Iida T., Takasawa T., Isobe K., Watanabe T., Itoh S., Yasutomi K., Kawahito S. (2012). A Low-Noise High Intrascene Dynamic Range CMOS Image Sensor with a 13 to 19b Variable-Resolution Column-Parallel Folding-Integration/Cyclic ADC. IEEE J. Solid-State Circuits.

[37] Pavelka J., Šikula J., Tacano M., Toita M. (2011). Activation Energy of RTS Noise. Radioengineering.

